# Extracellular Volume and Fibrosis Volume of Left Ventricular Myocardium Assessed by Cardiac Magnetic Resonance in Vaccinated and Unvaccinated Patients with a History of SARS-CoV-2 Infection

**DOI:** 10.1007/s12012-024-09929-3

**Published:** 2024-10-15

**Authors:** Paweł Gać, Wojciech Hajdusianek, Aleksandra Żórawik, Małgorzata Poręba, Rafał Poręba

**Affiliations:** 1 Centre of Diagnostic Imaging, 4th Military Hospital, Weigla 5, PL 50-981 Wroclaw, Poland; 2 Department of Environmental Health, Occupational Medicine and Epidemiology, Wroclaw Medical University, Mikulicza-Radeckiego 7, PL 50-368 Wroclaw, Poland; 3 Department of Paralympic Sports, Wroclaw University of Health and Sport Sciences, Witelona 25a, PL 51-617 Wroclaw, Poland; 4 Department of Angiology and Internal Medicine, Wroclaw Medical University, Borowska 213, PL 50-556 Wroclaw, Poland

**Keywords:** COVID-19, SARS-CoV-2, Vaccination status, Extracellular volume, Fibrosis volume, Cardiac magnetic resonance

## Abstract

Cardiac magnetic resonance (CMR) enables the assessment of tissue characteristics of the myocardium. Changes in the extracellular volume (ECV) and fibrosis volume (FV) of the myocardium are sensitive and early pathogenetic markers and have prognostic significance. The aim of the study was to assess ECV and FV of left ventricular myocardium in T1 mapping sequence in patients with a history of SARS-CoV-2 infection, considering vaccination status against COVID-19. The study group consisted of 97 patients (52.54 ± 8.31 years, 53% women and 47% men). The participants were divided into three subgroups: A) patients with a history of symptomatic SARS-CoV-2 infection, unvaccinated against COVID-19 (*n* = 39), B) patients with a history of symptomatic SARS-CoV-2 infection, with a full vaccination schedule against COVID-19 (*n* = 22), and C) persons without a history of SARS-CoV-2 infection constituting the control subgroup (C, *n* = 36). All patients underwent 1.5 T cardiac magnetic resonance. In subgroup A compared to subgroups B and C, both the ECV whole myocardium and ECV segments 2, 5–6, 8, and 10–11 were statistically significantly higher. In addition, the ECV segment 16 was statistically significantly higher in subgroup A than in subgroup C. Also, the FV whole myocardium was statistically significantly higher in subgroup A in comparison to subgroups B and C. There were no significant differences in ECV and FV between subgroups B and C. In summary, unvaccinated against COVID-19 patients with a history of symptomatic SARS-CoV-2 infection have higher myocardial ECV and FV values in the T1 mapping sequence, compared to those without COVID-19 and those suffering from COVID-19, previously vaccinated with the full vaccination schedule.

## Introduction

According to literature, severe acute respiratory syndrome coronavirus 2 (SARS-CoV-2) has affected more than 700,000,000 people across world [1, 2]. It has been reported that COVID-19 disease is associated with myocardial injury in about 17.85% of hospitalized cases [1]. SARS-CoV-2 infection may cause myocardial damage, among other effects, due to the associated immune response, hypercoagulable state with thrombotic events, and myocardial ischemia due to increased myocardial oxygen demand during infection (type 2 myocardial infarction) [2]. On the other hand, there are also reports about rare adverse event associated with COVID-19 vaccination – myocarditis with different prevalence depending on source [3, 4].

Heart can be assessed by cardiac magnetic resonance (CMR) imaging and one of the parameters is a volume of the myocardial tissue. It can be differentiated to cellular volume (myocytes) and extracellular volume (extracellular matrix) ECV, which composes about 6% of normal heart. An increase of ECV can be associated inter alia with an increase of myocardial collagen and further with heart interstitial fibrosis and therefore, it contributes to heart dysfunction [5–8]. It has also been studied that ECV can be used to predict cardiac recovery or remodeling after adverse events [9]. What is more, increased myocardial ECV obtained in computed tomography was associated with cardiac abnormalities caused by pulmonary embolism among patients with COVID-19 [10]. Finally, some articles suggest that ECV measurements may be as important as left ventricular ejection fraction (LVEF) parameter in cardiac imaging [6]. LVEF is a classic parameter for assessing the global systolic function of the left ventricle, ECV allows for the assessment of the tissue character of the whole myocardium, but also of individual segments of the left ventricular myocardium. In addition, changes in the tissue character of the myocardium are often a pathogenetic explanation for systolic dysfunction, in some cases preceding the occurrence of functional disorders. Both parameters have a high predictive value [11].

An increase in the amount of collagen in the heart can have various causes: it may be caused either by an increase in collagen (or other insoluble compounds such as amyloid protein) in the extracellular interstitium of the myocardium or by replacement of cardiomyocytes (e.g., after cells necrosis). Fibrosis can impair systolic and diastolic cardiac function. Fibrosis can significantly contribute to heart failure [12–14].

Previous studies of patients with COVID-19 have shown that 20–30% of them had cardiac troponin levels above the upper reference limit of the 99th percentile, which is an indicator of myocardial damage. Additionally, 50% of cardiac magnetic resonance (CMR) scans show typical pathologic findings, and out of these, 27% are compatible with myocarditis, 22% with ischemic heart disease, and the rest are nonspecific [3]. The assessment of ECV and FV enables precise identification of a group of patients with early, focal, but also diffuse injury of the left ventricular myocardium, which may cause regional and/or global left ventricular systolic dysfunction. This assessment is a quantitative assessment, comparable between patients, with high predictive value.

The aim of the study was to assess the extracellular volume and the fibrosis volume of left ventricular myocardium in T1 mapping sequence in patients with a history of SARS-COV-2 infection, considering vaccination status against COVID-19.

## Material and Methods

The study was conducted between July 2020 and December 2021. The following study protocol was adopted: in the first stage of the study, patients constituting the study group were recruited, i.e., patients with a history of symptomatic SARS-CoV-2 infection. The criteria for inclusion in the study group were age > 18 years, consent to participate in the study, and history of symptomatic SARS-CoV-2 infection (COVID-19 confirmed by PCR test). The exclusion criteria from the study included incomplete data on the history of COVID-19 disease, focal myocardial injury in the late gadolinium enhancement (LGE) sequence on cardiac magnetic resonance (CMR), and insufficient CMR image quality.

The size of the study group was estimated using a sample size calculator. The conditions for estimating the group size were as follows: population size: 3 million (population size of the Lower Silesian macroregion (Poland) in which recruitment was carried out), fraction size: 0.2 (maximum estimated prevalence of SARS-CoV-2 infection in the population at the time of recruitment [15]), maximum error: 10% and confidence level: 95% (standard values). The minimum required group size was 61. Based on the inclusion criteria, 72 patients were qualified for the project, and after excluding 9 patients from the study based on the exclusion criteria, they formed a study group of the required 63 people. Most patients from the study group were infected with the delta variant of the SARS-CoV-2 virus, which was dominant in the population in which the study was conducted during the study period. The study was completed before the date of confirmation of the first case of infection with the omicron variant of the SARS-CoV-2 virus in the study population. In the next stage, a control group was selected for the study group, patients without a history of SARS-CoV-2 infection, with similar anthropometric characteristics. The inclusion and exclusion criteria for the control group were analogous to those for the study group, except for the criterion of no previous symptomatic SARS-CoV-2 infection in the case of the control group. The control group was matched among patients under the care of the outpatient cardiology clinic, examined in our CMR laboratory, before the development of the COVID-19 epidemic, based on the matching criteria: analogous gender distribution, age, and BMI. 41 patients were initially classified into this group, who, after excluding 4 patients, formed a 37-person control group. During the study, a vaccination program against COVID-19 was launched. At this stage, patients who reported ambiguous data regarding vaccinations against COVID-19 in the anamnesis were excluded from the study groups (2 patients from the study group and 1 patient from the control group). The following were considered inconclusive data regarding vaccinations against COVID-19: patient’s refusal to provide information about participation in the vaccination program (in the case of 1 patient), incomplete basic vaccination cycle (in the case of 2 patients), and vaccination in a patient who has already had COVID-19 (in the case of 1 patient who also met the requirement for an incomplete vaccination course). In this way, in the third stage of the study, two groups of subjects were obtained: 61-person group with history of symptomatic SARS-CoV-2 infection and 36-person group without a history of SARS-CoV-2 infection. The study group (with history of symptomatic SARS-CoV-2 infection) was divided into 2 groups based on the criterion of vaccination against COVID-19 before symptomatic SARS-CoV-2 infection: 39 patients unvaccinated against COVID-19 and 22 patients with a full vaccination schedule against COVID-19 before symptomatic COVID-19.

As a result of the research protocol used, 97 patients finally participated in the study. The mean age of participants were 52.54 ± 8.31 years. In the study group, 53% of participants were women and 47% of participants were males. The participants were divided to three subgroups. The following subgroups were distinguished:

A. Patients with history of symptomatic SARS-CoV-2 infection unvaccinated against COVID-19 (*n* = 39).
B. Patients with a history of symptomatic SARS-CoV-2 infection, with a full vaccination schedule against COVID-19 (*n* = 22).
C. Patients without a history of SARS-CoV-2 infection (*n* = 36, control group).

The subgroups’ composition is presented in the Table 1 with the description of participants’ age, gender BMI, obesity, and prevalence of cardiovascular risk factors. The most common risk factor was arterial hypertension, whereas the least was diabetes.

**Table 1:** Clinical characteristics of the study subgroups

	Subgroup A (*n* = 39)	Subgroup B (*n* = 22)	Subgroup C (*n* = 36)	p
Age [years]^a^	52.21 ± 8.89	51.73 ± 9.63	52.81 ± 6.94	0.888
Gender^b^				0.883
Men	46.2	50.0	47.2	
Women	53.8	50.0	52.8	
BMI [kg/m^2^]^a^	26.07 ± 3.42	25.86 ± 3.09	24.77 ± 2.72	0.173
Overweight/obesity^b^				
Normal body mass	38.5	36.4	41.7	
Overweight	48.7	50.0	50.0	
Obesity	12.8	13.6	8.3	0.856
Coexistence of cardiovascular risk factors^b^				
Arterial hypertension	30.8	31.8	41.7	0.351
Type 2 diabetes	12.8	9.1	8.3	0.592
Dyslipidemia	25.6	18.2	22.2	0.788
Smoking	28.2	27.2	22.2	0.581

A—patients with a history of symptomatic SARS-CoV-2 infection, unvaccinated against COVID-19, B—patients with a history of symptomatic SARS-CoV-2 infection, with a full vaccination schedule against COVID-19, C—patients without a history of SARS-CoV-2 infection

*BMI* body mass index

^a^Quantitative variable expressed as mean ± standard deviation

^b^Categorical variable expressed as percentage

Cardiac magnetic resonance (CMR) was performed using one 1.5-T Magneton Aera device (Siemens Healthcare, Forchheim, Germany). An electrocardiography-gated breath-hold protocol was used. A bolus of 0.2 mmol/kg body weight of gadobutrol (Gadovist, Bayer Healthcare, Leverkusen, Germany) was administered intravenously into the antecubital fossa. Cine sequences, short tau inversion recovery (STIR) sequences, and late gadolinium enhancement (LGE) sequences, as well as T1 mapping sequences were performed. Cine imaging was performed in LV 2-, 3-, and 4-chamber apical views as well as in short-axis views encompassing the entire LV myocardium using balanced steady-state free precession (SSFP) gradient echo (slice/gap thickness: 10/0 mm, matrix: 256 × 192; in-plane resolution: 1.4 × 1.4 mm^2^; TR/TE: 3.0/1.3 ms, flip angle: 59°, parallel imaging technique (generalized autocalibrating partially parallel acquisition GRAPPA)). LGE imaging was performed using T1-weighted segmented inversion-recovery pulse sequence, 10 min after administration of the contrast agent. The following LGE sequence parameters were used: slice/gap thickness: 10/0 mm, matrix: 256 × 192, in-plane resolution: 1.4 × 1.4 mm^2^, TR/TE: 650/4.9 ms, flip angle: 30°, and inversion time set to null normal myocardium. T1 mapping sequences were obtained in end diastole in short-axis orientation in three slices (basal, midventricular, and apical). For myocardial T1 mapping, MOLLI (modified Look-Locker inversion) recovery acquisition scheme with motion correction (MOCO) was applied. The following T1 mapping sequence parameters were used: slice/gap thickness: 10/0 mm, matrix: 256 × 192, in-plane resolution: 1.4 × 1.4 mm^2^, TR/TE: 305.8/4.9 ms, and flip angle: 35°.

Extracellular volume of left ventricle (ECV) was calculated from native myocardial T1 time and post-contrast myocardial T1 time, native blood pool T1 time and post-contrast blood pool T1 time, and hematocrit values using standard mathematical formula 1. ECV is expressed in % [16–20].

1 $$ECV = \left( {1 - hematocrit} \right)\frac{{\left( {\frac{1}{{T1_{{myo\,post}} }} - \frac{1}{{T1_{{myo\,pre}} }}} \right)}}{{\left( {\frac{1}{{T1_{{blood\,post}} }} - \frac{1}{{T1_{{blood\,pre}} }}} \right)}} .$$

ECV—extracellular volume of left ventricle; T1 myo post—post-contrast myocardial T1 time; T1 myo pre—native (before contrast administration) myocardial T1 time; T1 blood post—post-contrast blood pool T1 time; and T1 blood pre—native blood T1 time.

The Fibrosis Volume of left ventricle (FV) was calculated by multiplying the ECV by the ratio of the left ventricular mass and the myocardial density (constant: 1.05 g/ml)—formula 2.

2 $$FV = ECV\, \times \,\frac{left\, ventricular \,mass}{{myocardial \,density}}.$$

ECV—extracellular volume of left ventricle; myocardial density is a constant 1.05 g/ml.

FV is expressed in ml. FV corresponds to the term found in other literature—*indexed extracellular volume or iECV* and is an adjustment of ECV for ventricular myocardial volume [21–23].

**Fig. 1:**
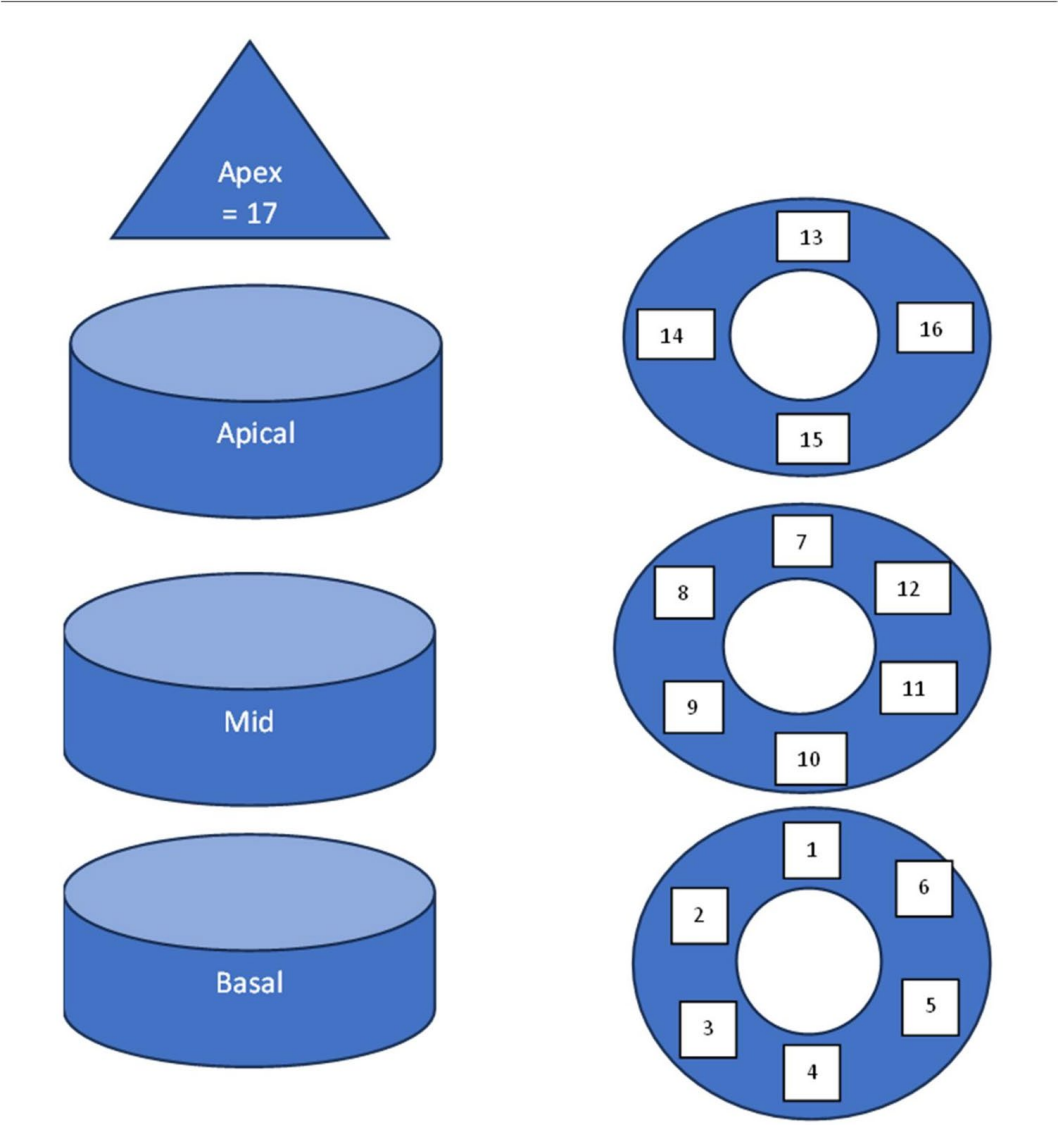
Diagram representing standardized American Heart Association’s myocardial segmentation model for heart imaging of left ventricle. On the left side, long-axis plane presenting division to basal, mid, and apical third of ventricle. On the right side, short-axis plane presents the division of each third to segments. 1, 7, 13: anterior. 2, 8: anteroseptal. 3, 9: inferoseptal. 4, 10, 15: inferior. 5, 11: inferolateral. 6, 12: anterolateral. 14: septal. 16: lateral. 17: apex

Mean ECV and mean FV were calculated of the whole myocardium and mean ECV was described in the 16-segment American Heart Association (AHA) myocardial model (see model description below). Segments 1–16 were used, but the apex, which is segment 17, was not included in the study [24, 25]. The division of the left ventricular myocardium into segments according to AHA is presented in Fig. 1.

Statistical analysis was conducted with Dell Statistica 13 Software (Dell Inc., Round Rock, TX, USA). Qualitative (categorical) variables are presented as percentages. Qualitative variables were compared using chi-square test. Quantitative variables are presented as means ± standard deviations and were analyzed in following manner. First, the variable distributions were studied with both histograms plotting with visual assessment and statistical test for distribution—Shapiro–Wilk test. In the case of normally distributed variables, ANOVA (one-way parametric) was used. In the case of variables with a non-normal distribution, the non-parametric equivalent of the analysis of variance, the Kruskal–Wallis ANOVA test was used. Statistically significant differences between arithmetic means were determined with the Newman–Keuls post hoc test. To determine the relationship between the studied variables, a correlation analysis was performed. In the case of quantitative variables with a normal distribution, Pearson’s r correlation coefficients were determined, and in the case of quantitative variables with a non-normal distribution, Spearman’s r coefficients were determined. The adopted level of statistical significance was 0.05.

## Results

All patients in subgroup A and B had symptomatic SARS-CoV-2 infection and none of patients of subgroup C had it. Mean duration of COVID-19 symptoms of patients of subgroup A was 20.84 ± 16.89 days and of patients of subgroup B was 8.26 ± 4.59 days. Most unvaccinated patients were hospitalized due to COVID-19 infection—about 87.2% people and only 9.1% of vaccinated patients. Furthermore, 13.9% of unvaccinated where admitted to intensive care unit with the mean duration of hospitalization about 22.48 ± 15.79 days. 88.9% patients from control subgroup C were vaccinated for COVID-19. The majority of both vaccinated and control subgroup received at least two doses of COVID-19 vaccination. For the people of subgroup B, the mean time between last dose of COVID-19 vaccine and symptoms of COVID-19 infections were 71.76 ± 39.42 days. The data are summarized in Table 2.

**Table 2:** Characteristics of SARS-CoV-2 infection and COVID-19 vaccination status of the study subgroups

	Subgroup A (*n* = 39)	Subgroup B (*n* = 22)	Subgroup C (*n* = 36)	*p*
Symptomatic SARS-CoV-2 infection^b^	100.0	100.0	0.0	A-B: 1.000 A-C: 0.000 B-C: 0.000
Duration of infection symptoms [days]^a^	26.18 ± 11.78	8.26 ± 4.59	N/A	A-B: 0.001 A-C: - B-C: -
Drugs^b^				
Remdesivir	2.5	9.0	N/A	A-B: 0.532 A-C: - B-C: -
Chloroquine	2.5	4.5	N/A	A-B: 0.807 A-C: - B-C: -
Antibiotics	35.9	13.6	N/A	A-B: 0.062 A-C: - B-C: -
Steroids	15.4	9.0	N/A	A-B: 0.485 A-C: - B-C: -
Hospitalization due to infection^a^	87.2	9.1	0.0	A-B: 0.005 A-C: 0.001 B-C: 0.332
Duration of hospitalization due to infection [days]^a^	20.84 ± 16.89	7.17 ± 6.48	N/A	A-B: 0.003 A-C: - B-C: -
Hospitalization in the ICU due to infection^b^	13.9	0.0	0.0	A-B: 0.219 A-C: 0.219 B-C: 1.000
Duration of hospitalization in the ICU due to infection [days]^a^	22.48 ± 15.79	N/A	N/A	A-B: - A-C: - B-C: -
Intubation during hospitalization in the ICU^b^	10.2	N/A	N/A	A-B: - A-C: - B-C: -
Vaccinations against COVID-19^b^	0.0	100.0	88.9	A-B: 0.000 A-C: 0.001 B-C: 0.346
1 dose of vaccination^b^	0.0	4.5	8.3	A-B: 0.283 A-C: 0.141 B-C: 0.583
2 doses of vaccination^b^	0.0	63.6	51.5	A-B: 0.001 A-C: 0.001 B-C: 0.387
≥ 3 doses of vaccination^b^	0.0	31.8	27.7	A-B: 0.002 A-C: 0.004 B-C: 0.751
Vaccine type				
Comirnaty (Pfizer-BioNTech)^b^	0.0	54.4	50.0	A-B: 0.001 A-C: 0.001 B-C: 0.756
Spikevax (Moderna, NIAID)^b^	0.0	18.2	13.8	A-B: 0.025 A-C: 0.054 B-C: 0.671
Johnson & Johnson vaccine (Janssen Pharmaceuticals)^b^	0.0	4.5	8.3	A-B: 0.283 A-C: 0.141 B-C: 0.583
Vaxzevria (AstraZeneca)^b^	0.0	22.7	16.7	A-B: 0.011 A-C: 0.032 B-C: 0.594
Time between last dose of vaccination and symptoms of infection [days]^a^	N/A	71.76 ± 39.42	N/A	A-B: - A-C: - B-C: -

A—patients with a history of symptomatic SARS-CoV-2 infection, unvaccinated against COVID-19, B—patients with a history of symptomatic SARS-CoV-2 infection, with a full vaccination schedule against COVID-19, C—patients without a history of SARS-CoV-2 infection

N/A not applicable, *ICU* intensive care unit

^a^Quantitative variable expressed as mean ± standard deviation

^b^Categorical variable expressed as percentage

The standard cardiac magnetic resonance parameters were studied, such as left ventricular ejection fraction, ventricular mass, or atrial area between all three subgroups. No statistically significant differences were found, and all details are shown in Table 3.

**Table 3:** Standard cardiac magnetic resonance parameters in the study subgroups

	Subgroup A (*n* = 39)	Subgroup B (*n* = 22)	Subgroup C (*n* = 36)	*p*
LAA [cm^2^]^a^	23.42 ± 3.43	23.69 ± 4.02	22.57 ± 3.78	0.271
RAA [cm^2^]^a^	20.96 ± 4.05	19.95 ± 3.84	21.16 ± 3.03	0.181
LVEDD [mm]^a^	57.11 ± 11.43	54.84 ± 8.62	55.48 ± 7.86	0.389
LVESD [mm]^a^	32.74 ± 10.09	33.93 ± 9.53	34.59 ± 8.11	0.437
aIVSDD [mm]^a^	7.86 ± 3.74	8.11 ± 4.02	7.96 ± 2.07	0.804
PWDD [mm]^a^	6.98 ± 3.03	6.43 ± 2.83	6.53 ± 2.42	0.470
LVMI [g/m^2^]^a^	84.17 ± 16.42	81.94 ± 16.71	82.42 ± 15.43	0.604
RVEDD [mm]^a^	38.48 ± 10.95	36.16 ± 9.93	37.59 ± 11.03	0.394
LVEF [%]^a^	67.02 ± 6.03	66.59 ± 5.48	67.42 ± 4.38	0.520
RVEF [%]^a^	58.14 ± 7.27	58.29 ± 8.49	56.78 ± 7.36	0.465
edema foci^b^	0.0	0.0	0.0	1.000
LGE foci^b^	0.0	0.0	0.0	1.000

A—patients with a history of symptomatic SARS-CoV-2 infection, unvaccinated against COVID-19, B—patients with a history of symptomatic SARS-CoV-2 infection, with a full vaccination schedule against COVID-19, C—patients without a history of SARS-CoV-2 infection

*aIVSDD* anterior interventricular septal diastolic diameter, *LVEF* left ventricular ejection fraction, *LAA* left atrial area, *LGE* late gadolinium enhancement, *LVEDD* left ventricular end-diastolic diameter, *LVESD* left ventricular end-systolic diameter, *LVMI* left ventricular mass index, PWDD posterior wall diastolic diameter, *RAA* right atrial area, *RVEDD* right ventricular end-diastolic diameter, *RVEF* right ventricular ejection fraction

^a^Quantitative variable expressed as mean ± standard deviation

^b^Categorical variable expressed as percentage

**Fig. 2:**
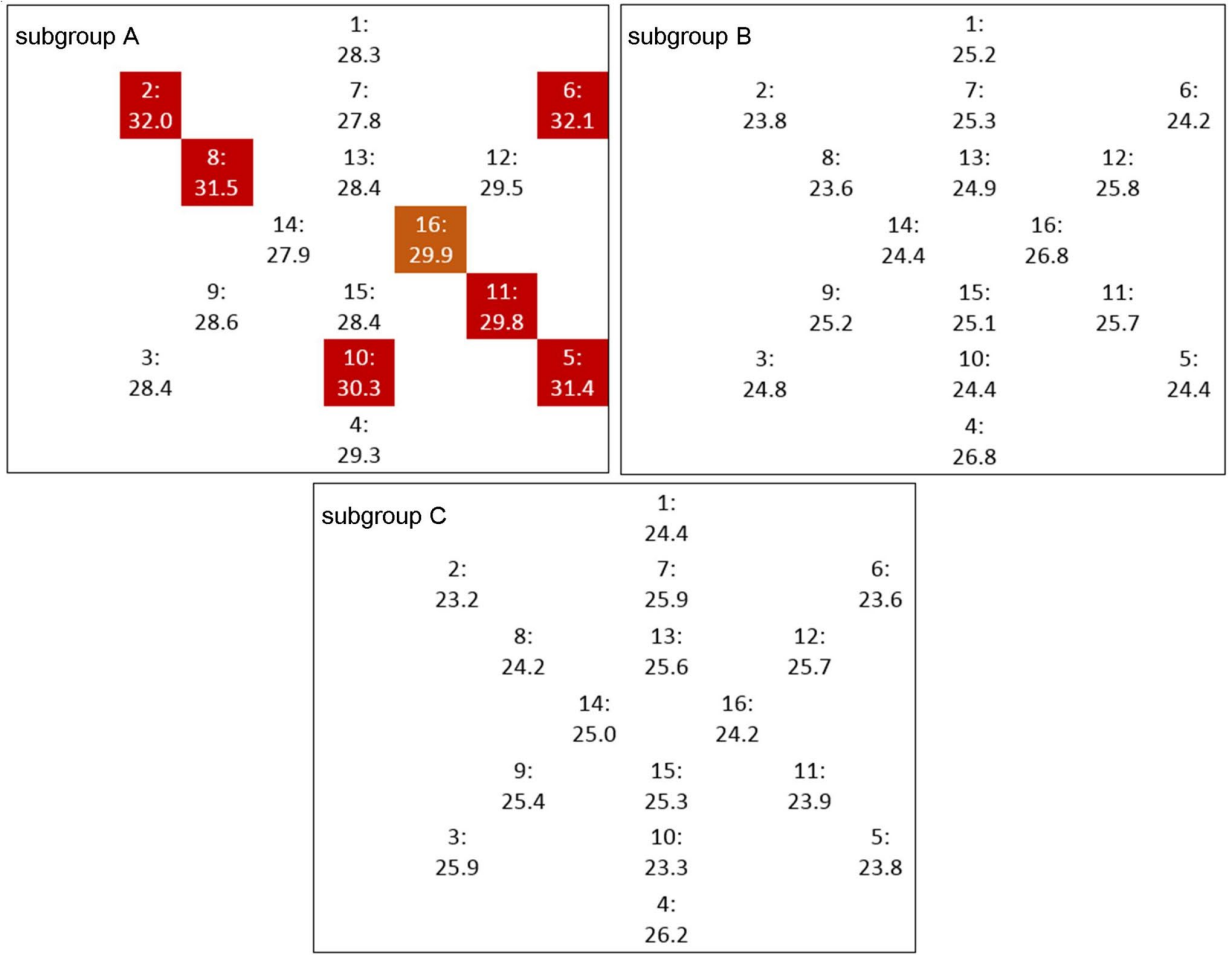
ECV measured in CMR on AHA cardiac imaging segment model. Numbers 1–16 in the upper part of each square are number of segments. Number in the lower part of each square is ECV value in (%). Red squares present significant differences (*p* < 0.05) between subgroup A and subgroups B and C. The orange square presents significant difference (*p* < 0.05) between subgroup A and subgroup C. The statistical significance of differences between groups was tested using the ANOVA (one-way parametric) analysis for normally distributed variables and using the Kruskal–Wallis ANOVA test for non-normally distributed variables. Individual differences were assessed by the Newman–Keuls post hoc test. *ECV* extracellular volume, *CMR* cardiac magnetic resonance, *AHA* American Heart Association

Analyzing extracellular volume (ECV) and fibrosis volume among subgroups the following differences were found. ECV of the whole myocardium and of basal layer of myocardium were significantly higher in patient of subgroup A compared to both subgroups B and C (29.87 ± 3.56 vs 25.13 ± 2.74 and 25.06 ± 2.16, *p* < 0.05; 30.25 ± 3.47 vs 24.86 ± 3.42 and 24.52 ± 2.84, *p* < 0.05; respectively). ECV of middle layer was significantly higher in subgroup A in comparison to subgroup C with 29.58 ± 2.62 vs 24.75 ± 2.62 *p* < 0.05. When comparing ECV among different segments of myocardium, significantly higher ECV in subgroup A in comparison to subgroup B and C in myocardium segments 2, 5, 6, 8, 10, and 11 were found and statically significant difference between subgroups A and C for segment 16 were obtained. Furthermore, analyzing fibrosis volume, significant difference between subgroups A and B and C with 32.01 ± 3.40 ml vs 24.44 ± 3.71 and 24.91 ± 2.03 was found (*p* < 0.05). Details are presented in Table 4 and Fig. 2. In further analysis, a Pearson’s correlation was conducted, and significant correlations are as follows: the duration of hospitalization in intensive care unit correlated with ECV whole myocardium and fibrosis volume whole myocardium (both *p* < 0.05 and *r* = 0.37 and 0.35, respectively). The time between last dose of vaccination and symptoms of infection correlated with ECV whole myocardium with *r* – 0.34 and *p* < 0.05. Table 5 presents the correlation analysis.

**Table 4:** Extracellular volume and fibrosis volume in the study subgroups

	Subgroup A (*n* = 39)	Subgroup B (*n* = 22)	Subgroup C (*n* = 36)	*p*
ECV whole myocardium [%]^a^	29.87 ± 3.56	25.13 ± 2.74	25.06 ± 2.16	A-B: 0.003 A-C: 0.002 B-C: 0.920
ECV basal layer [%]^a^	30.25 ± 3.47	24.86 ± 3.42	24.52 ± 2.84	A-B: 0.002 A-C: 0.001 B-C: 0.704
ECV middle layer [%]^a^	29.58 ± 2.62	25.01 ± 3.36	24.75 ± 2.62	A-B: 0.052 A-C: 0.002 B-C: 0.762
ECV apical layer [%]^a^	28.65 ± 4.42	25.21 ± 3.65	24.86 ± 4.03	0.061
ECV segment 1 [%]^a^	28.32 ± 4.48	25.20 ± 3.55	24.44 ± 3.87	0.059
ECV segment 2 [%]^a^	32.04 ± 2.57	23.77 ± 3.45	23.18 ± 3.62	A-B: 0.001 A-C: 0.001 B-C: 0.754
ECV segment 3 [%]^a^	28.37 ± 3.53	24.82 ± 4.13	25.87 ± 2.04	0.067
ECV segment 4 [%]^a^	29.26 ± 3.62	26.82 ± 4.18	26.19 ± 4.90	0.065
ECV segment 5 [%]^a^	31.45 ± 3.86	24.39 ± 2.83	23.82 ± 3.75	A-B: 0.002 A-C: 0.001 B-C: 0.706
ECV segment 6 [%]^a^	32.13 ± 2.50	24.17 ± 3.24	23.59 ± 3.08	A-B: 0.001 A-C: 0.001 B-C: 0.704
ECV segment 7 [%]^a^	27.84 ± 4.03	25.26 ± 3.46	25.90 ± 3.77	0.096
ECV segment 8 [%]^a^	31.47 ± 2.48	23.63 ± 2.57	24.17 ± 4.17	A-B: 0.001 A-C: 0.002 B-C: 0.754
ECV segment 9 [%]^a^	28.57 ± 3.16	25.18 ± 3.15	25.45 ± 3.14	0.058
ECV segment 10 [%]^a^	30.30 ± 3.76	24.40 ± 2.65	23.32 ± 3.42	A-B: 0.002 A-C: 0.001 B-C: 0.491
ECV segment 11 [%]^a^	29.78 ± 3.85	25.75 ± 3.24	23.90 ± 3.96	A-B: 0.003 A-C: 0.001 B-C: 0.262
ECV segment 12 [%]^a^	29.51 ± 2.02	25.81 ± 3.12	25.74 ± 3.43	0.061
ECV segment 13 [%]^a^	28.44 ± 4.63	24.88 ± 3.18	25.65 ± 4.89	0.066
ECV segment 14 [%]^a^	27.90 ± 4.67	24.40 ± 4.03	25.02 ± 3.55	0.063
ECV segment 15 [%]^a^	28.41 ± 2.72	25.14 ± 4.55	25.34 ± 4.09	0.071
ECV segment 16 [%]^a^	29.86 ± 3.13	26.85 ± 2.67	24.18 ± 2.42	A-B: 0.065 A-C: 0.002 B-C: 0.109
FV whole myocardium [ml]^a^	32.01 ± 3.40	24.44 ± 3.71	24.91 ± 2.03	A-B: 0.001 A-C: 0.001 B-C: 0.754

A—patients with a history of symptomatic SARS-CoV-2 infection, unvaccinated against COVID-19, B—patients with a history of symptomatic SARS-CoV-2 infection, with a full vaccination schedule against COVID-19, C—patients without a history of SARS-CoV-2 infection

*ECV* extracellular volume, *FV* fibrosis volume

^a^Quantitative variable expressed as mean ± standard deviation

**Table 5:** Correlations between variables characterizing SARS-CoV-2 infection versus extracellular volume and fibrosis volume in the study subgroups

	ECV whole myocardium [%]		FV whole myocardium [ml]	
	*r*	*p*	*r*	*p*
Duration of infection symptoms [days]	0.28	0.096	0.19	0.125
Duration of hospitalization due to infection [days]	0.25	0.098	0.28	0.097
Duration of hospitalization in the ICU due to infection [days]	0.37	0.039	0.35	0.041
Time between last dose of vaccination and symptoms of infection [days]	0.34	0.044	0.29	0.082

*ECV* extracellular volume, *FV* fibrosis volume, *ICU* intensive care unit

## Discussion

The subgroups studied did not differ in atrial area, ventricular diameter, or ventricular ejection fraction parameters. We found that myocardial extracellular volume and fibrosis volume were higher in unvaccinated patients with a history of symptomatic SARS-CoV-2 infection compared with vaccinated patients with infection and patients without SARS-CoV-2 infection. Moreover, we found that duration of ICU hospitalization correlates positively with both ECV and FV. We also obtained that the longer the time between the last dose of vaccine and the onset of COVID-19 symptoms, the higher ECV is.

The possible reasons for lower ECV and FV values in patients with SARS-CoV-2 infection vaccinated against COVID-19 may include an adequate immune response focused on the infection, a lower likelihood of a cytokine storm, lower imbalances in coagulation and hemolysis processes, and lower myocardial oxygen demand during infection [3]. As in our study, cardiac MRI has been widely used to assess the severity of myocardial damage, including the assessment of fibrosis—among other things, the available studies to date have shown that the presence of late gadolinium enhancement on cardiac MRI in hypertrophic cardiomyopathy reflects myocardial damage of an irreversible nature and is a strong predictor of subsequent complications [26, 27].

Studies conducted on animals—cats—have found that higher ECV is associated with hypertrophic cardiomyopathy and with diastolic dysfunction and left atrial size and increased interstitial fibrosis [28]. Similarly, the literature review supports ECV as a useful marker of myocardial disease associated with diffuse fibrosis [17]. Moreover, we found a one case report associating COVID-19 vaccine with retroperitoneal fibrosis [29]. ECV was previously found to be associated with abnormalities such as diabetes, heart failure, or aortic stenosis and may predict increased mortality [17, 30, 31]. From a clinical point of view, an increased ECV is not desirable. In one study, 60% of participants who recovered from COVID-19 that was severe or moderate had ECV increased after more than 90 days [32]. This result is partly consistent with our study—we also observed elevated ECV among patients after COVID-19. However, the study did not differentiate the participants’ vaccination status. There were rare reports of myocarditis after COVID-19 vaccination, in one case report, authors described the case of 30-year-old male hospitalized after second dose. A CMR performed eight weeks after vaccination showed myocardial fibrosis, which persisted on a follow-up CMR performed twenty-two weeks after vaccination [33]. Similarly, in other study it was reported that myocarditis after COVID-19 vaccination was connected to abnormal MRI results typical for myocarditis [34, 35]. In other research studying 20 patients with myocarditis after COVID-19 vaccination, an initial elevation of ECV was observed with decrease in the follow-up study. In research, global ECV with myocarditis was about 32.9% and 30.0% in follow-up study after 3 months [36]. In comparison, in our study mean global ECV of unvaccinated patients with COVID-19 was about 29.87% and 25.13% for vaccinated patients with COVID-19. It should be also emphasized that in systemic review myocarditis after COVID-19 vaccination was found to be affecting mainly men under 40 years old with highest risk between 12 and 24 [4]. In addition, it has been shown that patients with liver cirrhosis can develop breakthrough COVID-19 following full or partial vaccination. But these infections are associated with reduced mortality compared to SARS-CoV-2 infection in patients with liver cirrhosis not vaccinated against COVID-19 [37].

It should be noted that among patients who had symptomatic SARS-CoV-2 infection, in our study, patients who were unvaccinated against COVID-19 compared to patients who were vaccinated against COVID-19 had symptoms of infection longer and were also more often hospitalized due to COVID-19. Only patients not vaccinated against COVID-19 in our study required hospitalization in the intensive care unit. However, this should be interpreted with caution, because patients with mild symptoms may less often report to medical consultations and therefore remain unnoticed. Moreover, it should be emphasized that the selection of patients for the study was not completely random.

The strength of our study is the demonstration of the relationship between SARS-CoV-2 infection in patients not vaccinated against COVID-19, on the one hand, and the extracellular volume of the myocardium and the volume of fibrosis, on the other hand, in patients without focal myocardial damage resulting from myocarditis. The relationship between SARS-CoV-2 infection and focal myocardial damage resulting from myocarditis is now quite well documented. Our results may indicate that even in patients without focal post-inflammatory myocardial damage, SARS-CoV-2 infection in a patient not vaccinated against COVID-19 may result in higher ECV and FV values. Higher ECV and FV values can be considered as unfavorable predictors of the potential occurrence of cardiomyopathy in future, which may lead to the need for heart transplantation in the long term [38].

The current discussed study has several important limitations. First, the small size of the study group should be mentioned. A total of 97 people were examined, which, according to the sample size calculator, is sufficient to obtain reliable results. However, it would be difficult to separate groups differing in COVID-19 vaccination status in the control group not suffering from COVID-19. The lack of such a division that would enable comparisons of 4 groups is the basic limitation of our study. Too small number of patients without a history of SARS-CoV-2 infection not vaccinated against COVID-19 did not allow for comparison of patients with a history of SARS-CoV-2 infection, vaccinated against COVID-19 with patients with a history of SARS-CoV-2 infection not vaccinated against COVID-19, patients without a history of infection SARS-CoV-2 vaccinated against COVID-19, and patients without a history of SARS-CoV-2 infection not vaccinated against COVID-19. An equally important limitation is the method of selecting patients for the study. Purposive selection and subjective inclusion and exclusion criteria were used. Such a planned study does not allow generalizing characteristics regarding the incidence of COVID-19, its clinical course, and vaccination against COVID-19 to the general population. The study was designed to be reliable in verifying the hypothesis that was the aim of the study. The values of individual variables characterizing the course of the disease in our study group should not be translated into characteristics of the course of the COVID-19 pandemic in the general population. An incomplete course of vaccination against COVID-19, vaccination against COVID-19 after SARS-CoV-2 infection and focal myocardial damage in CMR examination may be considered as questionable in the exclusion criteria from the study. The first two were determined by the size of the group. In the study group, if these situations were included, we would have single cases of patients presenting these special situations. It would be difficult to obtain statistically valid results. The exclusion of patients with focal left ventricular myocardium injury resulted from the aim of the study, which focused on the extracellular volume and fibrosis volume. The period during which patients were qualified for the project should also be considered an important limitation of the study. The study was conducted from July 2020 to December 2021. This study period resulted in the absence of infections caused by the omicron type of SARS-CoV-2 virus. Such a study period also resulted in recruitment for the study overlapping with the start of the COVID-19 vaccination program, which resulted in modifications to the objectives and method of conducting the study described in the study protocol. The lack of confirmation of the type of virus causing COVID-19 is also a drawback of our study. Finally, it should be added that the heterogeneity of the studied groups with respect to the characteristics of COVID-19 can also be considered a factor that may limit the statistical power of the obtained results. Separate groups of patients with a history of SARS-CoV-2 infection, vaccinated, and unvaccinated against COVID-19 differed in the frequency of hospitalization, the severity of the disease, and, to some extent, also in the treatment used. It should also be emphasized that the study conducted is an observational study with a 1-point assessment of effect markers. Further follow-up of the study group seems to be necessary.

## Conclusion

Patients unvaccinated against COVID-19 with a history of symptomatic SARS-CoV-2 infection have higher myocardial ECV and FV values in the T1 mapping sequence compared to those without symptomatic COVID-19 and those suffering from COVID-19 who were previously vaccinated with the full vaccination schedule. Higher ECV and FV values may be the result of mild myocardial inflammation during COVID-19 in this group of patients.

## Data Availability

The data presented in this study are available upon request from the corresponding author. The data are not publicly available.
